# Social dimensions as resources in promoting academic well-being: the case study of the University of Foggia

**DOI:** 10.3389/fpsyg.2024.1347532

**Published:** 2024-03-28

**Authors:** Fulvio Signore, Ciro Esposito, Immacolata Di Napoli, Barbara Agueli, Emanuela Ingusci, Terri Mannarini, Giusi Antonia Toto, Caterina Arcidiacono, Stefania Fantinelli

**Affiliations:** ^1^Department of Humanities, Letters, Cultural Heritage and Educational Studies, University of Foggia, Foggia, Italy; ^2^Department of Humanities, University of Naples “Federico II”, Naples, Italy; ^3^Department of Social Sciences, University of Naples “Federico II”, Naples, Italy; ^4^Department of Human and Social Sciences, University of Salento, Lecce, Italy

**Keywords:** well-being in academia, organizational identification, place attachment, territorial well-being, SEM, job resources

## Abstract

Recently, scholars have focused more on changes in higher education, leading to significant insights into the working lives of academics and certain related processes, such as stress or well-being. The interest in academia is also justified by the role of universities as institutions that promote health and well-being, serving as a bridge between society, the world of work, and the local community. This study aims to identify social factors that can enhance the well-being of academic workers (lecturers and technical–administrative staff), highlighting how processes linked to social identity, based on the dynamics of identification with a territory or an organization, can serve as resources that promote well-being. Researchers conducted the survey on 198 workers at the University of Foggia (South Italy). Correlation and reliability assessments were first performed between the variables. Finally, a SEM study was completed. The goodness of fit of the model seems to be sufficient. The social aspects examined in the study, namely, organization identification, territorial well-being, and place attachment, were positively and significantly correlated with general well-being. Findings of the study demonstrated that for teaching and technical–administrative staff, among the key components for enhancing well-being in the academic setting was the social dimension of relationships, understood both inside and outside the university. Therefore, acting the belonging process to an area, implementing and strengthening relations with the social actors involved, as well as on the sense of belonging and identification with an organization, can have precise impact in enhancing well-being.

## 1 Introduction

In recent years, more and more attention has been paid to changes in higher education, stimulating increasingly crucial insights into the working lives of academics and certain linked processes, such as stress or well-being (Brondino et al., 2022; Tilak and Kumar, 2022). The interest in academia is also supported by the fact that universities, as institutions, foster and enhance health and well-being, and institutions hold great significance in the minds of individuals and social groups, serving as a link between society, the world of work, and well-being of the local (Smith, 2007).

A significant role has been played, in this respect, by the present study on healthy organizations, in which emphasis is placed on specific elements that can enhance employees’ well-being. Healthy organizations, in actuality, are ones in which the combined presence of a good psychological climate and good practices allows for the creation of an environment that promotes the health and well-being of workers, in addition, of course, to the overall effectiveness of the organization (Di Fabio, 2017; Lowe, 2020). Within such organizations, in fact, the goal is to protect the business (Grawitch and Ballard, 2016) but by leveraging the inextricable relationship that exists between organizational performance and the well-being of workers (Arnoux-Nicolas et al., 2016), without creating an imbalance between the two. Recently, universities have also been the subject of studies on healthy organizations. Healthy universities would therefore represent a crucial strategy in approaching health-promoting environments (Dooris and Doherty, 2010). As an institution, higher education plays an important role in shaping society, with significant social, economic, and environmental landscape on a local, national, and international scale (Brennan et al., 2004).

Together with these factors, the increased interest in university well-being was also the result of the changes induced by the COVID-19 pandemic, which caused significant transformations in the academic environment, resulting in an increased complexity in the various social actors involved: academic activity is characterized by different stakeholders (faculty, administrative staff, students), all of whom have felt the effects of this massive transformation. The intricate shift of work in the academy has been demonstrated by several studies: the research by Casacchia et al. (2021) conducted on lecturers revealed that the use of new technologies on the one hand contributed to psychological issues but at the same time encouraged the ability to put themselves on the line, as well as personal growth in other participants. Additionally, new methods of working have increased uncertainty and instability in the workplace, changing times and modes, and consequently, impacting workers’ well-being (Evanoff et al., 2020; Pacheco et al., 2020). Universities therefore continue to encounter significant obstacles, leading to raised levels of work-related stress for academic, research, technical, and administrative staff (Tilak and Kumar, 2022) consider empowering these stakeholders as one of the priorities to invest in, in order to preserve the best aspects of educational institutions. For these reasons, a focus on the health of workers in universities became increasingly important. Indeed, although some studies point out that the higher education sector is currently evidencing strong interest in promoting a culture of well-being (Marcus et al., 2018; Amaya et al., 2019), little literature still focuses on this topic. Brooker and Woodyatt (2019) explored various aspects of wellbeing in higher education, including student and staff support, strategic leadership, and psychological wellbeing literacy. Tay (2021) further emphasized how research into factors that promote wellbeing in higher education can be an integral part of community wellbeing, thus mainly emphasizing the social aspect. In summary, prior research concentrated on either interactional or situational dimensions (e.g., family/home stressors, job insecurity), while there are no studies examining the individual dimension and perception in relation to social identity within the framework of work-organizational context. The latter aids in defining a person’s affective and belonging characteristics that can potentially stimulate energies and advance resources.

Because of the abovementioned context, this study aims to identify social factors that can support a broader understanding of academic workers’ well-being (e.g., lecturers and technical–administrative staff), highlighting how processes related to social identity in workers, based on the dynamics of identification with a territory or an organization, can trigger well-being. The research was conducted in the University of Foggia, in South Italy. The University of Foggia represents a very significant role for the area, not least because of the problems that have always plagued it, including organized crime. To this purpose, in reality, successive heads and rectors have sought to establish vibrant communities, involving different stakeholders, to outline the university’s aims. From these moments of reflection, the main goals of the university for the benefit of the region emerged, including teaching, research and internationalization, building and infrastructure, spin-offs, business accelerators, start-ups and placement, social responsibility, sustainability and the environment, health, building and infrastructure, orientation and teacher training, culture, youth policy and sports, student services, organizational well-being and governance models, and press and public engagement. The role of the university for the territory was further reaffirmed by Head of State Mattarella, who speaking in attendance at the opening of the previous academic year emphasized the significance of forming consciences to combat organized crime, and in this regard, the University of Foggia is playing a central role for the territory. All institutions must work in synergy to offer Foggia a different future. The university, in actuality, fosters the cultural dynamism and zeal required to combat crime.

Within this context, then, protecting the well-being of the academic community, in terms of workers, is essential to the idea of offering a supportive foundation in the university’s operation in the area.

In this regard, therefore, this study aims to demonstrate how specific factors of social identification (with the university, with the territory, and with Foggia) can serve as catalysts to enhance academic well-being.

## 2 Theoretical foundations and hypothesis development

### 2.1 Social identity as a driver for well-being: the role of place attachment

Social identity is “that part of the self- concept that derives from a person’s sense of belonging to a group (or to a particular social group) and that is associated with the emotional and evaluative significance that derives from such belonging” ((Tajfel, 2010), p. 63). According to Tajfel (2010), social identity serves a crucial purpose for the self, since it contributes to retaining a healthy degree of personal self-esteem: in actuality, by introducing (positive) traits linked with those groups to which the person attaches significance, the person develops a “good” self-image. It is a crucial component of individual well-being, according to several research, including those by Eyles and Williams (2008) and (Rollero and De Piccoli, 2010). Social identity, however, is not only formed through groups, but also through physical places, especially places where people spend large amounts of their lives. Place identity (Proshansky, 1983) represents belonging defined by locality, by sharing the same space with a social group: therefore, individuals choose, attach to, and identify with certain places to enhance or maintain a positive social identity, just as they choose, attach to, and identify with a plurality of social groups (professional, political, ethnic, religious, etc.). In conclusion, places are an important source of identity content (Breakwell, 1999); they represent symbols and are invested with social connotations. Among places, cities have a special status, to the extent of structuring in their inhabitants an actual urban identity (Lalli, 1992). Indeed, being born in each city, or living there for significantly long periods, means absorbing traits and images, anthropological and psychological qualities that, while associated with the city, extend to its residents. These characteristics, transferring from the perception of the city to that of its people, become part of their social identity.

The affective component of place-related identity is defined through the concept of attachment, which denotes an affective link between an individual and a particular place, the main characteristic of which is the person’s tendency to maintain proximity to it (Hidalgo and Hernández, 2001). Even more explicitly than the cognitive aspects of identity, attachment to place contributes to determining the level of existential pleasure of individuals; in actuality, those who possess a strong attachment bond seem to be happier with their lives than people without one (Bonnes et al., 2016). Additionally, the global COVID-19 pandemic significantly altered the way in which people developed connections to their place, also rekindling attachment to it, partly because of the mandatory lockdown and closure (Devine-Wright et al., 2020). Given the absence of a more thorough examination of the connection between this construct and overall well-being, the initial research hypothesis is as follows:

*H1:* Place attachment improves general academic workers’ well-being.

### 2.2 Social identity in the work context: organizational identification

Similar to the territory, the social identity that is created in connection to the work context, i.e., the degree of identification that people develop with their work organization, also favors the triggering of processes linked to well-being. Organizational identification is defined as “the conception of belonging to a group, as well as the value and emotional significance attributed to belonging to that group” (Smidts et al., 2001). Numerous investigations have demonstrated that this social identification dynamic has a significant impact on well-being-related outcomes. Among these, the study by Welander et al. (2017) highlighted how, in different types of workers, social identification is an important variable mediating the relationship between personal and organizational resources, as defined according to the job demands-resources model. Indeed, social identity theory has also included affective components, such as the emotional significance and feelings of belonging that an individual gives to belonging, in its theorizations (Nordhall et al., 2020) found that within a group of teachers, processes related to social identity, based on cognitive mechanisms (processes such as incorporation and assimilation into a new social group) and emotional mechanisms (attachment/belonging/closeness, pride, esteem), are essential for enhancing mental health and work motivation. The study by Simbula et al. (2023) confirmed how, in line with theoretical conceptualizations of social identity and social exchange, factors such as identification and social relationships are significant in determining work-related well-being outcomes such as engagement. The study was carried out on workers in a longitudinal method; consequently, it appears that this position is also significant from a different angle in terms of time periods. The aforementioned studies indicate that phenomena related to social identity are also capable of explaining processes of well-being improvement, providing evidence for their potential investigation and analysis as antecedents (rather than mediators) of overall well-being. Put differently, they would be viewed as employment resources.

Furthermore, employees who strongly identify with their work are more likely to be dedicated to it (Loi et al., 2014; Karanika-Murray et al., 2015). Individuals therefore feel more personally invested and tend to achieve more satisfactory outcomes, both individually and for the organization through performance (Carmeli et al., 2007). Thus, according to the foregoing, the second research hypothesis is as follows:

*H2:* Organizational identification has a positive impact on general academic workers’ well-being.

### 2.3 The role of the university in fostering the well-being of academic workers

Universities, as higher institutions, include in their mission the cultural and social advancement of the communities in which they operate. These aspects, in fact, come under the so-called third mission, which parallels research and instruction. The main goal of the third mission is to convey societal principles by directly involving external actors (Cassella, 2017). The goal is therefore to “feed the local system […] promoting projects of innovation and change in the territory, transferring services and models of development of new technologies, activating at the same time feedback processes, which transform the university itself, making it more sensitive to the problems and needs of companies and/or a territory” (Lazzeroni et al., 2009). This concept thus emphasizes how the university establishes itself as a catalyst of regional growth. They therefore serve as a catalyst for the expansion of the interface between university and territory. Some studies acknowledged this leading, improving role for universities (Benneworth and Jongbloed, 2010; Lazzeroni and Piccaluga, 2015), by concentrating on the growth of the knowledge economy of their host region (Stachowiak et al., 2013) highlight the critical role of the social component in strengthening the interaction between the university and the local setting. The authors stress the value of the spaces of interaction and social or personal networks that might form in academic settings, factors that can trigger an increase in trust between the university and the local community. According to Goldstein and Glaser (2012), universities have significant capacity for supporting the development of their local community (economic, social), regardless of the importance of teaching, research, and technological advancement. This position becomes even more crucial in the presence of increasingly competitive and knowledge-based economies.

Additionally, as also noted by Solari and Gambarotto (2014), a feeling of connection and belonging to a place might enhance well-being. Specifically, it is the sense of rootedness that determines the development of a sense of identity, which can then lead to implications related to well-being. This need, therefore, can also extend to workers as a component of their own well-being. This idea was later adopted by Torrisi and Pernagallo, (2022), who in their research determined the role of territoriality in academic well-being as critical. Specifically, by connecting the construct of territoriality [examined in terms of (i) differences with foreign countries and (ii) multi-local] with work engagement and job satisfaction, it emerged that a higher sense of territorial well-being is associated with better job engagement and satisfaction.

As therefore explained above, many studies in the literature have concentrated on the welfare role represented by universities toward their host areas. In this study, we want to investigate the hypothesis that this role of universities is positively seen by the workers within it, thus fostering well-being. As mentioned earlier, in fact, social identity theory claims that an identification with the organizations to which one belongs might lead to the introjection of good traits associated with them to which the person attaches value, developing a positive self-image. The territorial development mission of universities, therefore, could allow workers to incorporate this positive component into their being, implementing their self-esteem and increasing individual well-being. Thus, the third hypothesis is as follows:

*H3:* The university’s capacity to enhance territorial well-being has a positive impact on academic workers’ well-being.

**Figure 1:**
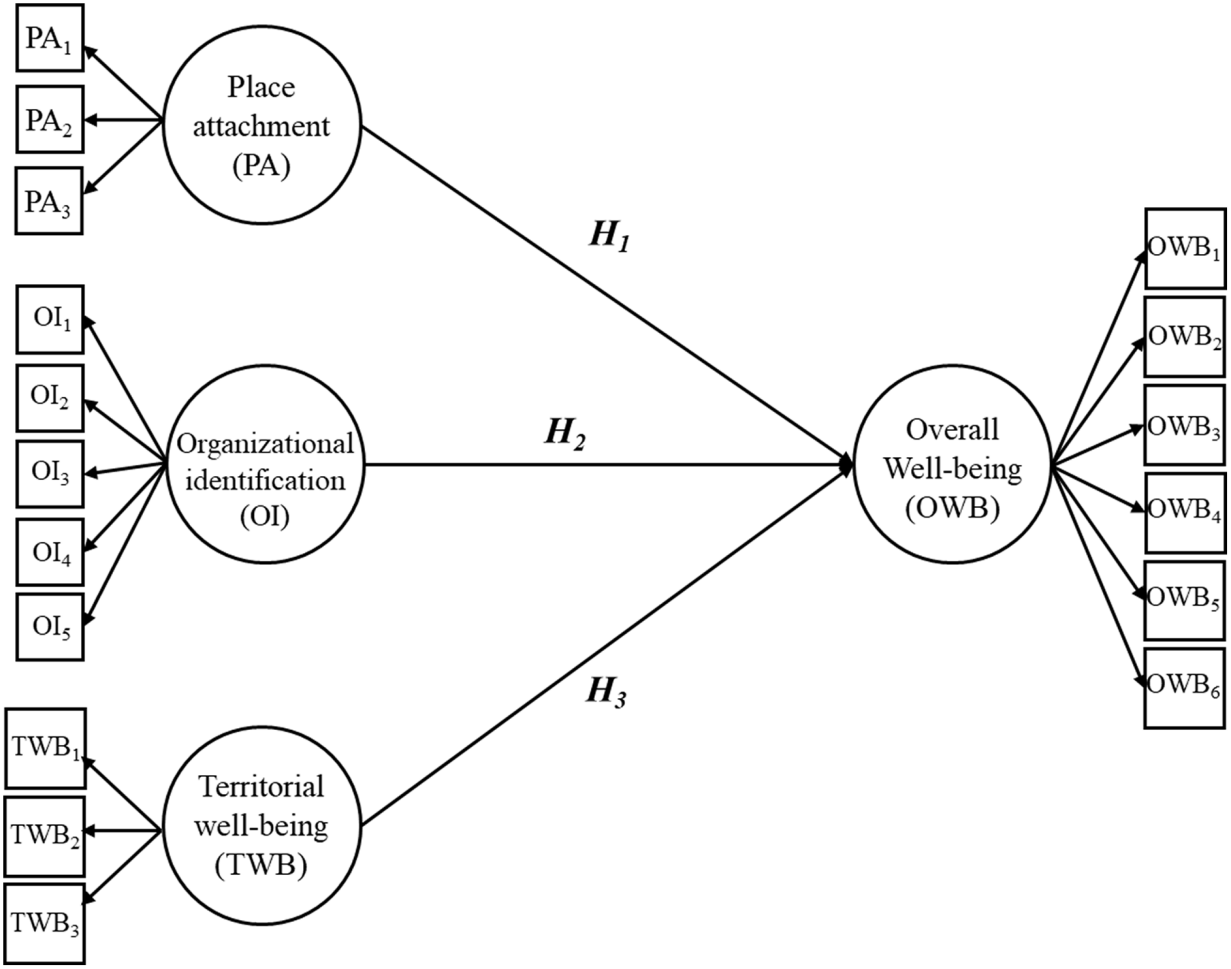
Full model along with research hypotheses.

Figure 1 displays the entire model along with research hypotheses.

## 3 Method

### 3.1 Procedure

The survey was conducted on 198 workers in the University of Foggia (South Italy). Regarding academic position, the sample consisted of lecturers (associate professors, full professors, TDa and TDb researchers – 134 individuals, 67.7%) and technical–administrative personnel (64 individuals, 32.3%). Research participants received a link through which they could fill in a questionnaire examining factors related to academic well-being. For participation, anonymity was assured and the possibility of withdrawing from the study at any time: the information was examined in aggregate form and without the possibility of tracing it back to the person in any scenario. The experiment was approved by the Ethics Committee for Psychological Research of the University of Foggia (protocol n. 36,714). Participants provided informed consent at the start of the online questionnaire completion form. The responses to the questionnaire were correctly gathered using the browser used to develop it and then properly tagged for further examination. The only sensitive data needed is that described in the participating section. The study is part of a larger project, called Pro.Be, distinguished by a multi-method (qualitative–quantitative) survey aimed at gathering the opinions of workers and students at the university, on a range of factors that can define the quality of organizational life and well-being in the academic setting of the University of Foggia. Apart from the University of Foggia, the initiative involves the collaboration of the University of Salento and the University Federico II of Naples.

### 3.2 Participants

Not all study participants responded to the demographic inquiries. Among the present responses, in sociodemographic terms, 57.9% of the sample were female workers (66 individuals), 39.5% were male workers (45 individuals), and 2.6% were nonbinary workers (3 individuals). 42.4% of the sample did not respond to the question (84 individuals). The predominant attributes expressed as a percentage are listed below: regarding marital status, 71.7% of the participants were married/cohabiting (81 individuals), 16.8% single (19 individuals), 10.6% separated/divorced (12 individuals), and 0.9% widowed (1 individual). 39.5% of the participants reported that they had underage children (45 individuals), 36.0% that they had no children (41 individuals), 16.7% that they had children of full age (19 individuals), and 7.9% that they had children of both full age and underage (9 individuals).

Lastly, almost 96.4% of the participants said that they lived in the university’s hometown or province (102 individuals), 0.9% in other parts of the regions (1 individual), 3.6% in regions bordering the university (4 individuals), and 4.5% in distant regions (5 individuals). 43.2% of the sample did not respond to the question (86 individuals).

### 3.3 Analysis procedures

The empirical model was validated in its assumptions utilizing structural equation modeling. Prior to using this approach, certain preliminary analyses were created to validate the dependability and internal consistency of the measurements. To this end, reliability assessments such as Cronbach’s alpha, McDonald’s omega, and average variance extracted were performed. Additionally, to assess the theoretical consistency between the constructs, correlation tests were performed between the variables, calculated using synthetic indicators. Lastly, a structural equation model was evaluated, with particular attention paid to the goodness of fit of the structural and measurement model and the related fit indices (RMSEA, SRMR, CFI, TLI). Analyses were done using Jamovi program (Rosseel, 2019; Gallucci and Jentschke, 2021; Jamovi Project, 2021; R Core Team, 2021).

### 3.4 The survey and variables

The academic staff intercepted for research purposes answered a standardized quantitative questionnaire characterized by several constructs already validated in the literature. The items mentioning place demonstrate clearly to the in-depth case study and concentrate on the city of Foggia, as evident in the constructs below:

• Place attachment: three items from (Boley et al., 2021) and translated in Italian. Example item is “I am very attached to Foggia.” The response is coded on a Likert scale that assumes choice options ranging from 1 = Not at all to 6 = Totally.

• Organizational identification: five items from (Mael and Tetrick, 1992), as translated in Italian by Brondino et al. (2022). Example item is “The success of the university is mine too.” The reaction is measured on a Likert scale assuming choice options ranging from 1 = Not at all agree to 6 = Completely agree.

• Territorial well-being: three *ad hoc* constructed items. Example of item is “In your opinion, does the University of Foggia contribute to the overall well-being of the area, in terms of the cultural, social, and economic wealth it directly or indirectly brings?.” The response is coded on a Likert scale which assumes choice options ranging from 1 = Not at all to 6 = Very much.

• Overall well-being: six items derived from the present scores on well-being as hypothesized by Esposito et al. (2022b), i.e., interpersonal, occupational, health, community, psychological, economic, and overall. An example of an item is “Given how your life is in general these days, which number do you choose now?.” The response is coded on a Likert scale that assumes choice options ranging from 1 = Worse to 10 = Better.

## 4 Results

Table 1 shows that the values for skewness and kurtosis were all within the range − 2/ +2 and − 7/ +7, respectively (Curran et al., 1996); consequently, the distribution of the data appears apparently normal.

**Table 1:** Descriptive statistics of the sample.

	Place attachment	Organizational identification	Territorial well-being	Overall well-being
Mean	3.53	4.98	4.48	7.44
Standard deviation	1.42	1.17	0.861	1.71
Skewness	-0.129	-1.87	-1.24	-0.806
Std. error skewness	0.219	0.212	0.219	0.222
Kurtosis	-0.822	3.55	3.22	1.01
Std. error kurtosis	0.435	0.422	0.435	0.440

The variables considered in the study all had outstanding reliability and validity indices, as Table 2 demonstrates, with the only exception of the variable territorial well-being, which reveals a slightly lower Cronbach’s alpha than the norm (0.66). Although the latter turns out to be the sole measure of reliability that is slightly subthreshold (in particular, an acceptable McDonald’s omega of 0.73 and an Average Variance Extracted (AVE) over 0.50), it opens up the possibility of further in-depth research of the items of territorial well-being in the future. Regarding the remaining constructs, McDonald’s omega is above 0.80 for the other constructs, whereas AVE is above 0.5 for all of them.

**Table 2:** Reliability indices for study variables.

Variables	*α*	*ω*	AVE
Place attachment	0.923	0.927	0.810
Organizational identification	0.897	0.882	0.633
Territorial well-being	0.660	0.726	0.508
Overall well-being	0.887	0.894	0.561

Regarding correlations (Table 3), conducted using synthetic indicators, data demonstrate positive and significant correlations between organizational identification and territorial well-being (*r*_1_ = 0.426, <0,001), place attachment (*r*_2_ = 0.342, <0.001), and overall well-being (*r*_3_ = 0.445, <0.001), as well as between territorial well-being and place attachment (*r*_4_ = 0.258, <0.001) and overall well-being (*r*_5_ = 0.437, <0.001), and place attachment and overall well-being (*r*_6_ = 0.324, <0.001).

**Table 3:** Correlation matrix between variables.

	Organizational identification	Territorial well-being	Place attachment	Overall well-being
Organizational identification	–			
Territorial well-being	0.426***	–		
Place attachment	0.342***	0.258**	–	
Overall well-being	0.445***	0.437***	0.324***	–

** < 0.01 *** < 0.001.

Moreover, in terms of the structural equation model, the following standards were deemed suitable: (i) comparative fit index (hereafter CFI) ≥0.90, root–mean–square error of approximation (hereafter RMSEA) ≤0.08, and standardized root–mean–square residuals (hereafter SRMR) ≤0.10 (O’Boyle and Williams, 2011).

**Figure 2:**
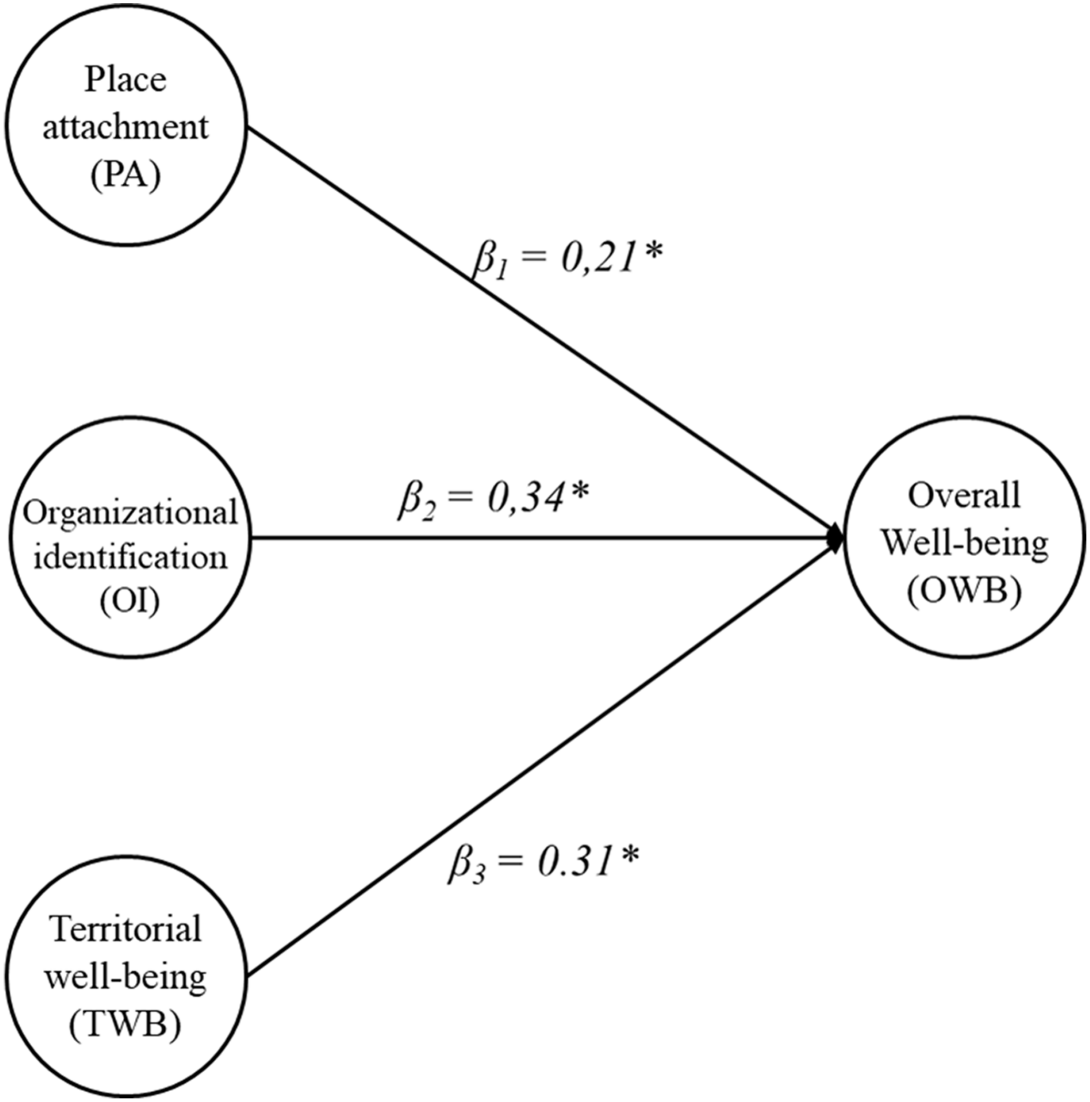
Model estimates.

Hence, the goodness of fit of the model appears to be satisfactory except for RMSEA index, as CFI = 0.91, TLI = 0.90, SRMR = 0.075, and RMSEA = 0.089. The value of RMSEA, however, as indicated by Kenny et al. (2015), can be significantly impacted by the low sample size, so much so that the authors themselves raise the question of whether it should be utilized with small numbers. Regarding the structural model, all variables are sufficiently and significantly measured by their manifest indicators, as the saturation ranges for the factor organizational identification from 0.632 to 0.938, for territorial well-being from 0.433 to 0.923, for place attachment from 0.832 to 0.948, and for overall well-being from 0.464 to 0.907 (Table 4). As far as the measurement model is concerned, conversely, the explicit theoretical conjectures appear to be supported (as can be seen in Figure 2). Specifically, in fact, the place attachment (*β*_1_ = 0.21, <0.05), the organizational identification (*β*_2_ = 0.34, <0.05), and the role of territorial well-being that the mission of the university itself promotes in the area (*β*_3_ = 0.31, <0.05) appear to be viewed by the academic staff as helpful tools to improve well-being, understood in its multidimensional dimension. Thus, hypotheses H1, H2, and H3 were verified.

**Table 4:** Factor loadings and latent variables.

Latent variables	Indicators	Factor loadings	*p*.value
Organizational Identification	Item 1	0.819	< 0.001
	Item 2	0.708	< 0.001
	Item 3	0.937	< 0.001
	Item 4	0.888	< 0.001
	Item 5	0.634	< 0.001
Territorial well-being	Item 1	0.946	< 0.001
	Item 2	0.563	< 0.001
	Item 3	0.416	< 0.001
Place attachment	Item 1	0.912	< 0.001
	Item 2	0.947	< 0.001
	Item 3	0.832	< 0.001
Overall well-being	Item 1	0.549	< 0.001
	Item 2	0.844	< 0.001
	Item 3	0.751	< 0.001
	Item 4	0.501	< 0.001
	Item 5	0.846	< 0.001
	Item 6	0.721	< 0.001

## 5 Discussion

Universities are among the significant institutions for the development of future generations of workers and for the social integration function they fulfill for the communities in which they are situated (Smith, 2007). To fulfill these missions, they need to study the organizational and psychological framework that enables workers to enhance their quality of life and well-being (Brondino et al., 2022). Universities, in turn, as they have undergone numerous changes recently, including the COVID-19 health emergency, require careful consideration and research to determine potential risk and protective variables (Esposito et al., 2022a). The current study aimed to close a gap in the existing literature, which focused on either interactional or situational dimensions (e.g., family/home stressors, job insecurity) that may be related to well-being, while there are no studies examining the individual dimension and perception in relation to social identity. Furthermore, studies in the field of social psychology demonstrate how social identity-related constructs (such as place attachment, organizational identification, and territorial well-being) act as antecedents of general well-being, contrary to what has been theorized in the job demands-resources model, which rather viewed them as mediators. Consequently, it appears intriguing to consider them as antecedents in the frame of work-organizational context. Overall, this study confirms how organizational identification, i.e., that individual process allows individuals (or workers) to incorporate favorable aspects of their organization within their own identity structure, acts as a driver of well-being, as identified in other studies, such as that of Hameed et al. (2022) and De Giorgio et al. (2023) At the same time, the study emphasizes other aspects related to relationality (in a broad sense) are also significant in promoting activities related to well-being. Specifically, in fact, as stated in the studies of Joaquim Araújo De Azevedo et al. (2013), Scannell and Gifford (2017), the significant relationship with one’s place (city) is extremely important in determining aspects of well-being. Lastly, although there are still few studies on the subject (Torrisi and Pernagallo, 2022), the possibility of intervening in the territory, promoting the growth of the economy and structures, can also instill well-being processes in workers.

Findings of the study demonstrated that for teaching and technical–administrative staff, among the key components for enhancing well-being in the academic setting is the social dimension of relationships, understood both inside and outside the university. As previously mentioned, relationships are the foundation for the development of a social identity (Harris and Orth, 2020) that is linked both to working groups and to the territory itself. Actually, the process by which this identity is realized is based on the logic of forming and strengthening interactional bonds with social groups based on belonging to a place or territory. Acting on the sense of belonging to an area, implementing and improving relations with the social actors involved, as well as on the sense of belonging and identification with an organization, can have specific effects in enhancing well-being.

Lastly, the study suggests a possible application of the identification elements associated with a location or an organization in terms of enhancing motivational processes, thus directly related to well-being. Specifically, according to the standpoint of the JD-R model (Bakker et al., 2023), these components could be considered the human and organizational resources. Personal resources are defined as “positive self-evaluations that are linked to resiliency and refer to individuals’ sense of their ability to control and impact upon their environment successfully (…) [and] (a) are functional in … protect from threats and the associated physiological and psychological costs, and (b) stimulate personal growth and development” (Xanthopoulou et al., 2009, p. 236). Although this suggestion requires further study, with larger and more structured samples, the findings of this research demonstrate how attachment to a place and organizational identification, which might well be caused by the introduction of positive traits derived from the social mission of the university’s third mission (i.e., from fostering the social well-being of the community), can enhance workers’ well-being.

The study findings highlight the significance of creating well-being-oriented intervention programs that involve community and territorial engagement. Regular evaluations of the organizational climate could be instrumental in maintaining and advancing the identified protective variables.

## 6 Limitations

The study has some limitations that must be considered when interpreting the findings. First is the numerosity of the sample, which does not allow the findings to be generalized. Therefore, future research could use a bigger sample in order to reinforce the findings, going beyond the limitations of the present study, which probably also impacts specific fit indices such as the RMSEA. In this sense, therefore, an extension of the sample could lead to more consistent findings. Second, the metric of territorial well-being has a marginally lower reliability than desired by the relevant indices. This factor needs to be considered when interpreting the findings and in potential follow-up research, which might use more rigorous and reliable methods. Additionally, being a case study, the research requires additional data to support the findings, using workers from other universities in addition to the one considered in the study. Third, the measures in the study are derived from self-report questionnaires and are therefore susceptible to potential bias; a follow-up study using a more rigorous research design and a more thorough psychometric analysis of the constructs (particularly for the *ad hoc* built items) supported by the utilization of more objective measurements (e.g., number of events in the local territory which originate from the university, count of university–territory interactions) could give strength and empirical validity to the findings of this initial study. Finally, the nature of the study is cross-sectional, which is why a longitudinal extension of it could provide both a deeper knowledge of the phenomenon being studied and a better robustness of results.

## 7 Conclusion

The study highlighted further that it is crucial for the university institution to address specific societal issues. Given the positive contribution of place attachment, organizational identification, and territorial well-being in fostering well-being among workers in the academic setting, it is crucial to consider this aspect as a potential element in raising the quality of life of workers. The social actors that define the university setting, in fact, can profit from these phenomena of a social nature for the development of positive personal self-esteem through the introduction of (positive) traits associated with groups or territories to which the individual attributes value. The human being, in reality, can develop a good self-image through social interactions or belonging to a common context or territory (Breakwell, 1999), elements that then flow into identity structures helpful for a coherent consideration of one’s being. In this particular case, in fact, individual factors such as well-being turned out to be associated with processes intrinsically tied to the territory (place attachment and territorial well-being) and to a shared sense of belonging (organizational identification), all of which can serve as the foundation for the formation of social identities. Thus, the interaction between workers to strengthen organizational identification and the territory to create a sense of belonging constitutes work resources, i.e., components capable of creating motivational processes and favorable results. According to Ryff’s theory (Ryff, 1989) and application studies in this area (Haslam et al., 2009; Sharma and Sharma, 2010), if social groups or belonging to a territory conveys positive meanings, purposes, and affiliations, these can influence psychological implications of improved well-being.

### 7.1 Practical implications

Ultimately, this study, despite its limitations, offers an empirical insight on the way in which workers’ well-being can be promoted. Organizations, in actuality, are frequently seen as a straightforward means of generating wealth and advantage, thus without considering the relational and social aspect established inside. In this sense, in fact, an enhanced ability for dialog with the territory, promoting mutual learning and development, may inadvertently impact worker well-being. Therefore, focusing on the positive aspects of the organizations and the places in which they operate could allow a better identification with the worker and, consequently, greater well-being and quality of life in the organization. At the same time, finally, encouraging collaboration between territories and institutions, such as universities, highlighting their function as “promoters of well-being,” may benefit workers.

## Data availability statement

The original contributions presented in the study are included in the article, further inquiries can be directed to the corresponding author, stefania.fantinelli@unifg.it.

## Ethics statement

The studies involving humans were approved by the University of Foggia Committee Board. The studies were conducted in accordance with the local legislation and institutional requirements. The participants provided their written informed consent to participate in this study. Written informed consent was obtained from the individual(s) for the publication of any potentially identifiable images or data included in this article.

## Author contributions

FS: Conceptualization, Data curation, Formal analysis, Investigation, Methodology, Resources, Software, Supervision, Validation, Visualization, Writing – original draft, Writing – review & editing. CE: Conceptualization, Data curation, Formal analysis, Investigation, Methodology, Resources, Software, Supervision, Validation, Visualization, Writing – original draft, Writing – review & editing. IN: Conceptualization, Data curation, Investigation, Methodology, Project administration, Resources, Software, Supervision, Validation, Visualization, Writing – original draft, Writing – review & editing. BA: Conceptualization, Data curation, Formal analysis, Investigation, Methodology, Software, Supervision, Validation, Writing – original draft, Writing – review & editing. EI: Conceptualization, Data curation, Formal analysis, Investigation, Methodology, Software, Supervision, Validation, Writing – original draft, Writing – review & editing. TM: Conceptualization, Data curation, Investigation, Methodology, Resources, Software, Supervision, Validation, Writing – original draft, Writing – review & editing. GT: Conceptualization, Data curation, Funding acquisition, Investigation, Methodology, Project administration, Resources, Software, Supervision, Validation, Writing – original draft, Writing – review & editing. CA: Conceptualization, Data curation, Formal analysis, Investigation, Methodology, Resources, Software, Supervision, Validation, Visualization, Writing – original draft, Writing – review & editing. SF: Conceptualization, Data curation, Formal analysis, Investigation, Methodology, Resources, Software, Supervision, Validation, Visualization, Writing – original draft, Writing – review & editing.
